# Ontogeny of Melatonin Secretion and Functional Maturation of the Pineal Gland in the Embryonic Turkey (*Meleagris gallopavo*)

**DOI:** 10.3390/ani15233437

**Published:** 2025-11-28

**Authors:** Magdalena Prusik

**Affiliations:** Department of Histology and Embryology, Faculty of Veterinary Medicine, University of Warmia and Mazury in Olsztyn, Oczapowskiego Str. 13, 10-719 Olsztyn, Poland; mprusik@uwm.edu.pl; Tel.: +48-89-523-34-49

**Keywords:** pineal organ, light, melatonin, oscillator, norepinephrine, turkey, birds, embryo, in vitro, superfusion culture

## Abstract

The pineal gland is a key component of the avian circadian system, responsible for melatonin (MLT) secretion that reflects and regulates daily rhythmicity. While the post-hatching turkey is known for its highly sensitive and efficient pineal system, the functional development of this gland during embryogenesis has not been described. To determine the measurable onset and regulation of MLT secretion, pineal glands from turkey embryos aged 22, 24, and 26 days (ED 22–26) were incubated in a superfusion culture under various light conditions and with or without norepinephrine (NE). MLT concentrations in the medium were measured by radioimmunoassay. Under the experimental conditions, MLT secretion was first reliably measurable at ED 22 and increased thereafter toward hatching. Under a 12L:12D cycle, pinealocytes exhibited a stable circadian rhythm with a fourfold amplitude between night and day. The glands adapted to a reversed light–dark cycle and responded strongly to a 3 h nocturnal light pulse with a 30% decrease in MLT levels. Rhythmic MLT secretion persisted under constant light or darkness, indicating an active endogenous oscillator. NE markedly suppressed MLT secretion in all embryos, confirming the early development of adrenergic regulation. Thus, by ED 22 the embryonic turkey pineal exhibits photosensitivity, endogenous rhythmicity, and NE responsiveness, although secretion at earlier stages cannot be excluded due to detection limits. These results fill a gap in understanding avian pineal ontogeny and establish the turkey embryo as a good model for studying the early development of circadian regulation in birds.

## Simple Summary

The pineal gland is a key neuroendocrine organ responsible for the production of melatonin (MLT), which regulates circadian rhythms in birds. Although the turkey is known for its exceptionally light-sensitive pineal gland after hatching, little has been known about its function during embryonic development. This study shows that, under the employed superfusion culture and assay conditions, melatonin secretion in turkey embryos was first reliably measurable on the 22nd day of incubation (ED 22), at which point the pineal gland is already capable of responding to light and norepinephrine and generating an endogenous circadian rhythm. These findings show that the turkey pineal gland reaches functional maturity before hatching and provide a valuable model for studying the development of biological rhythms in birds.

## 1. Introduction

The avian pineal organ is a neuroendocrine gland that produces the principal hormone melatonin (MLT) at night, exhibits photosensory properties, and can autonomously generate a circadian rhythm of MLT secretion. Among all vertebrates, the pineal gland of post-hatching birds displays the greatest species diversity in both morphology and in the mechanisms regulating MLT biosynthesis and secretion. Histologically, the avian pineal parenchyma occurs in several histological forms—saccular, tubulo-follicular, solid-follicular, and solid—and each species presents a characteristic combination of features within these categories. This morphological variety is mirrored by species-specific differences in control of MLT release [1,2,3,4,5,6,7,8,9,10,11,12,13,14,15,16,17,18,19,20].

The developmental morphology of the avian pineal gland has been described in detail in the chicken—at the histological and cellular levels—and in the turkey—at both histological and three-dimensional levels. In turkeys, the parenchyma remains purely follicular throughout the embryonic period and through the first post-hatching year, without the parafollicular cells that form the solid parenchymal type in chickens; in chickens, solid parenchyma appears already during embryogenesis [21,22,23,24]. Four stages of embryonic pineal development have been identified in the turkey: (1) linear growth of the pineal canal with rosette-lined walls doubling in length every two days from ED (embryonic day) 4 to ED 8; (2) rapid growth between ED 8 and ED 10 with emergence of small round follicles; (3) inhibited longitudinal growth from ED 10 to ED 20 with an increase in follicle number and size; and (4) gradual elongation from ED 20 to hatching with onset of cellular differentiation within growing follicles [24].

In the chicken embryo, the gland grows rapidly until ED 12 and then slows markedly until hatching, while the parenchyma progressively transforms from follicular to solid and pinealocytes differentiate [22,25,26]. These developmental differences underscore distinct trajectories of maturation and control of MLT secretion between species.

The ontogeny of pineal physiology is coordinated: phototransduction, circadian oscillation, and neuroendocrine output emerge on overlapping but non-identical timelines [27,28,29,30]. In birds, the pineal functions both as an intrinsic circadian pacemaker and as a photosensitive, melatonin-secreting organ; however, developmental trajectories remain unevenly documented across taxa. Pioneering work in chicken and goose embryos showed that rhythmic MLT release can precede full retinal maturation and that adrenergic input modulates output late in incubation [3,20,28,31,32,33]. Melatonin biosynthesis begins during embryonic life, as demonstrated in vivo and in vitro in chicken, goose, and Japanese quail; embryonic MLT secretion is rhythmic (higher at night, lower during the day), light-dependent, and paced by an endogenous oscillator. Notably, interspecific differences have been reported in the timing of MLT onset, rhythm profiles and amplitude, photosensitivity, and oscillator activity [3,27,28,29,30,31,32,33,34,35,36,37,38,39,40,41,42,43,44,45].

MLT secretion in birds is also modulated by norepinephrine (NE), the terminal neurotransmitter in the pathway linking retina and hypothalamus to the pineal. NE acts via α_2_-adrenoceptors to inhibit MLT release in avian pinealocytes [46]. Sympathetic innervation is present at least three days before hatching in quail, and NE inhibits daily MLT secretion in goose embryos but not in chicken embryos [3,33,47].

By contrast, comparable functional data for turkeys are scarce and fragmented [7,8,14,15]. Turkeys are an important agricultural species with robust pre- and post-hatch photoperiodic responses, making them a relevant model for understanding how early pineal function may shape perinatal physiology and behavior. Yet the precise embryonic onset of autonomous circadian rhythmicity, photic entrainability, and adrenergic responsiveness in turkey pineal tissue has not been established under controlled conditions. In this study, I used superfused embryonic turkey pineal glands sampled at clearly defined embryonic stages to quantify basal and stimulated MLT secretion.

I tested whether, at the onset of measurable secretion, the embryonic turkey pineal already exhibits three functional features characteristic of post-hatching tissue: (i) day–night rhythmicity with light entrainment, (ii) an active endogenous oscillator, and (iii) norepinephrine-mediated inhibition. This work addresses a defined gap in avian developmental chronobiology and provides a practical framework for timing and assessing functional maturation within Galliformes.

## 2. Materials and Methods

### 2.1. Embryos

Fertilized eggs of the domestic turkey (*Meleagris gallopavo*) (total number = 84) were obtained from a local breeding farm and incubated in an Ova-Easy 580 Advance Series II cabinet incubator (Brinsea Products Inc., Titusville, FL, USA) at 37.5–38.0 °C and 55–60% relative humidity, with automatic turning of the eggs around the long axis by 45° every 30 min. The incubation was carried out under a 12 h light:12 h dark cycle (photoperiod 07:00–19:00). During the light phase, the eggs were illuminated with white LED light (5000 K) emitted from lamps mounted on the external surface of the translucent incubator door. The light intensity measured on the egg surface ranged from 15 to 20 lx. During the dark phase, incubation was conducted in complete darkness. All experimental procedures on embryos were performed in accordance with Polish and European Union regulations on animal experimentation.

### 2.2. In Vitro Study

#### 2.2.1. Culture Medium

Medium 199 containing Earle’s salts and HEPES (Sigma-Aldrich, St. Louis, MO, USA) was prepared from powder according to the manufacturer’s instructions, adjusted to pH 7.2 with NaOH, and sterilized by filtration. Prior to use, sterile ascorbic acid solution (Sigma-Aldrich, St. Louis, MO, USA) and Antibiotic-Antimycotic Solution (Sigma-Aldrich, St. Louis, MO, USA) were added to achieve final concentrations of 300 mg/L ascorbic acid, 100 IU/mL penicillin, 100 µg/mL streptomycin, and 0.25 µg/mL amphotericin B. A freshly prepared 1 mM norepinephrine bitartrate solution (Sigma-Aldrich, St. Louis, MO, USA) was sterilized by filtration and diluted in the culture medium to a final concentration of 10 µM.

#### 2.2.2. Superfusion Culture Technique

Decapitation of ED 22, ED 24 and ED 26 turkey embryos was performed between 09:00 and 11:00. Pineal organs were isolated immediately after decapitation, wrapped in nylon mesh, and placed individually in culture chambers connected to a multichannel peristaltic pump (Cole Parmer Instrument Company, Vernon Hills, IL, USA) and a manual fraction collector via the upper ports. The culture medium, continuously equilibrated with a gas mixture of 95% O_2_ and 5% CO_2_, was perfused through the lower port of each chamber at a rate of 0.05 mL/min. Incubation was conducted at 38.5 °C in a water bath (JULABO GmbH, Seelbach, Germany). During the photophase, the chambers were illuminated with white fluorescent light (100 lx at the chamber surface). During the scotophase, lightproof polyethylene covers were placed over the chambers. The superfusion system enabled simultaneous incubation of 24 explants, each in a separate chamber.

### 2.3. Experimental Design

Three independent experiments lasting from 1 to 3 days were designed to examine (I) the effect of light conditions, (II) the activity of the endogenous oscillator, and (III) the influence of norepinephrine on MLT secretion.

Samples of culture medium were collected into consecutively numbered tubes at 30 min intervals throughout each experiment: from 14:30 on day 1 to 11:30 on the final day for 3-day experiments, and from 14:30 to 06:30 for 1-day experiments. Samples were immediately frozen at −20 °C. After completion of all experiments, MLT concentrations in the medium were determined by radioimmunoassay (RIA), and time-course MLT secretion profiles were plotted for each experiment and embryonic age at every sampled time point.

I examined three embryonic ages—ED 22 (youngest), ED 24 (intermediate), and ED 26 (oldest); hereafter ‘all embryonic stages’ refers to these three ages.

#### 2.3.1. Experiment I—Effect of Light

Pineal organs from 22-, 24-, and 26-day-old embryos were incubated for three days under a 12 h light:12 h dark cycle (12L:12D; photophase 07:00–19:00, scotophase 19:00–07:00; group I) or a reversed dark-light cycle (12D:12L; photophase 19:00–07:00, scotophase 07:00–19:00; group II). A third group (group III) was incubated under the 12L:12D cycle for one day, and during the night the explants were exposed to white light (100 lx at the chamber surface) between 01:00 and 04:00. Each experimental series included explants from all age groups and treatments, with 36 pineal organs in total (4 per age/treatment combination).

#### 2.3.2. Experiment II—Activity of the Endogenous Oscillator

Pineal organs from 22-, 24-, and 26-day-old embryos were incubated for three days under continuous darkness (24D; group IV) or continuous light (24L; group V). The experiment consisted of three identical replicates with respect to embryo age and protocol, comprising 24 pineal glands in total (4 per age/treatment combination).

#### 2.3.3. Experiment III—Effect of Norepinephrine

In group VI, pineal organs from 22-, 24-, and 26-day-old embryos were cultured for one day under a 12L:12D cycle, followed by two days in continuous darkness. During the second and third subjective days (07:00–19:00), the explants were incubated in medium containing 10 µM NE. Group VII explants were cultured under a 12L:12D cycle for one day and exposed to NE (10 µM) during the night between 01:00 and 04:00. The experiment consisted of four identical replicates for each age group and treatment, with 24 pineal organs in total (4 per age/treatment combination).

### 2.4. Analytical Procedures

#### 2.4.1. Chemicals

The anti-melatonin antibody Prospect 6C was kindly provided by Dr. Andrew Foldes (Division of Animal Production, CSIRO, Blacktown, Australia). [3H]-melatonin was purchased from PerkinElmer (Waltham, MA, USA), gelatin from Merck (Billerica, MA, USA), and toluene from POCH (Gliwice, Poland). High-purity chemicals were obtained from J.T. Baker Chemicals (Center Valley, PA, USA) and used to prepare mobile phases: sodium acetate, sodium dihydrogen phosphate, disodium EDTA, 1-octanesulfonic acid sodium salt, citric acid, acetic acid, and phosphoric acid. Methanol and acetonitrile (gradient-grade HPLC purity) were purchased from Merck Millipore (Billerica, MA, USA). 5-HTOL was obtained from Santa Cruz Biotechnology (Dallas, TX, USA). All other reagents were purchased from Sigma-Aldrich (St. Louis, MO, USA). Ultrapure water (18.2 MΩ cm, TOC ≤ 3 ppb) was freshly prepared using a Milli-Q^®^ IQ 7003/05 purification system (Merck Millipore, Billerica, MA, USA) and used in all procedures.

#### 2.4.2. Melatonin Radioimmunoassay

MLT concentrations in medium samples were determined by direct radioimmunoassay (RIA) using the Prospect 6C antiserum and [3H]-melatonin (3.2 TBq/mmol), as previously described and validated [14]. The assay volume ranged from 40 to 400 µL, depending on the age-related differences in MLT secretion. The assay sensitivity was <10 pg/tube, and intra- and inter-assay coefficients of variation were <10%.

#### 2.4.3. Statistical Analysis

Data from Experiment I were analyzed using repeated-measures analysis of variance (ANOVA), with treatment as the main factor and sampling time as the repeated measure. The least significant difference (LSD) test was applied as a post hoc comparison. Data from Experiment II were analyzed using one-way ANOVA followed by Duncan’s multiple range test. Differences were considered statistically significant at *p* < 0.05.

All statistical analyses were performed using Dell Statistica, version 13.1 PL (Dell Inc., Tulsa, OK, USA).

## 3. Results

### 3.1. Experiment I—Effect of Light

#### 3.1.1. Group I—12L:12D Cycle

**Figure 1:**
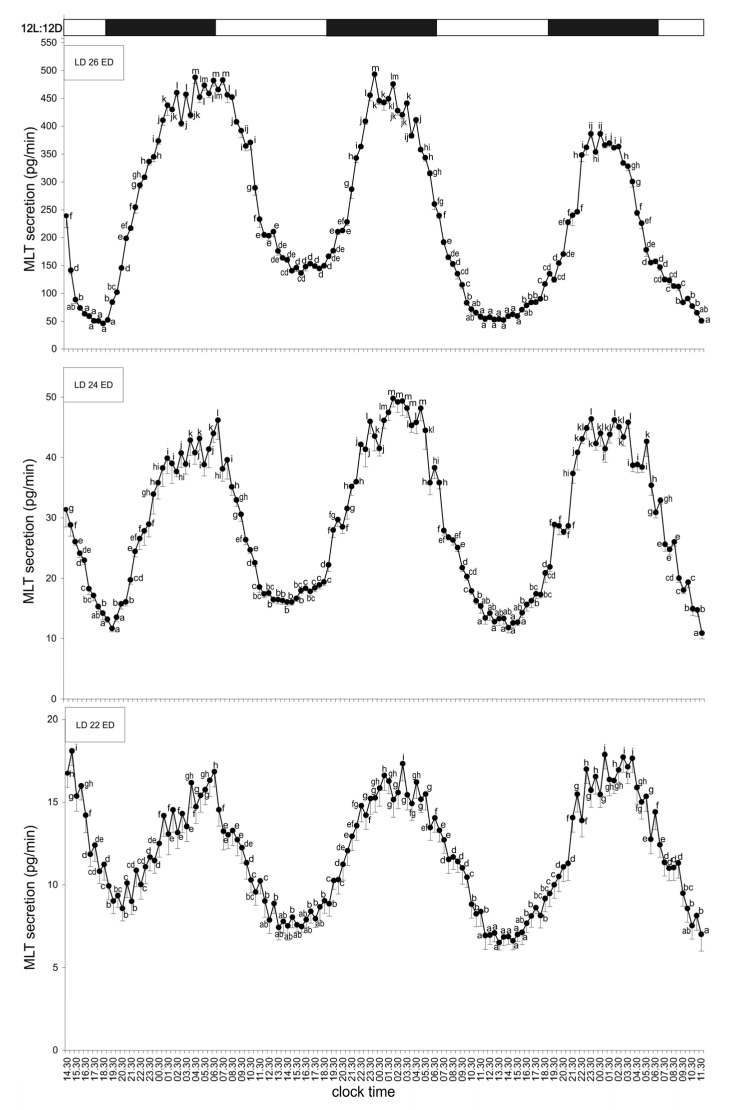
Secretion of melatonin (MLT; mean ± SEM) from the pineal organs of turkey embryos at embryonic days (ED) 22–26, incubated for three days under a 12L:12D light–dark cycle (light phase from 07:00 to 19:00). Different letters indicate significant differences between time points within each age group, while identical letters denote means that are not significantly different. MLT secretion in all age groups followed a similar rhythmic pattern; however, pronounced differences in the overall secretion levels were observed among the groups.

Pineal organs from all studied embryonic stages, incubated under a 12 h light:12 h dark cycle (light phase 07:00–19:00) secreted MLT in a regular diurnal rhythm throughout the experiment. During the first day, MLT secretion increased stepwise after the onset of scotophase, reached its maximum between 23:30 and 07:30, and then gradually decreased. The lowest MLT levels were observed between 15:00 and 18:30. In the following days, both the peak and trough phases were shorter, occurring between 23:30 and 04:00 and between 13:00 and 15:30, respectively. The amplitude of the rhythm remained approximately fourfold throughout the experiment. The MLT concentration in ED 26 embryos was about ten times higher than in ED 24 embryos, and the levels in ED 24 embryos were three to four times higher than in ED 22 embryos (Figure 1).

#### 3.1.2. Group II—12D:12L Cycle

**Figure 2:**
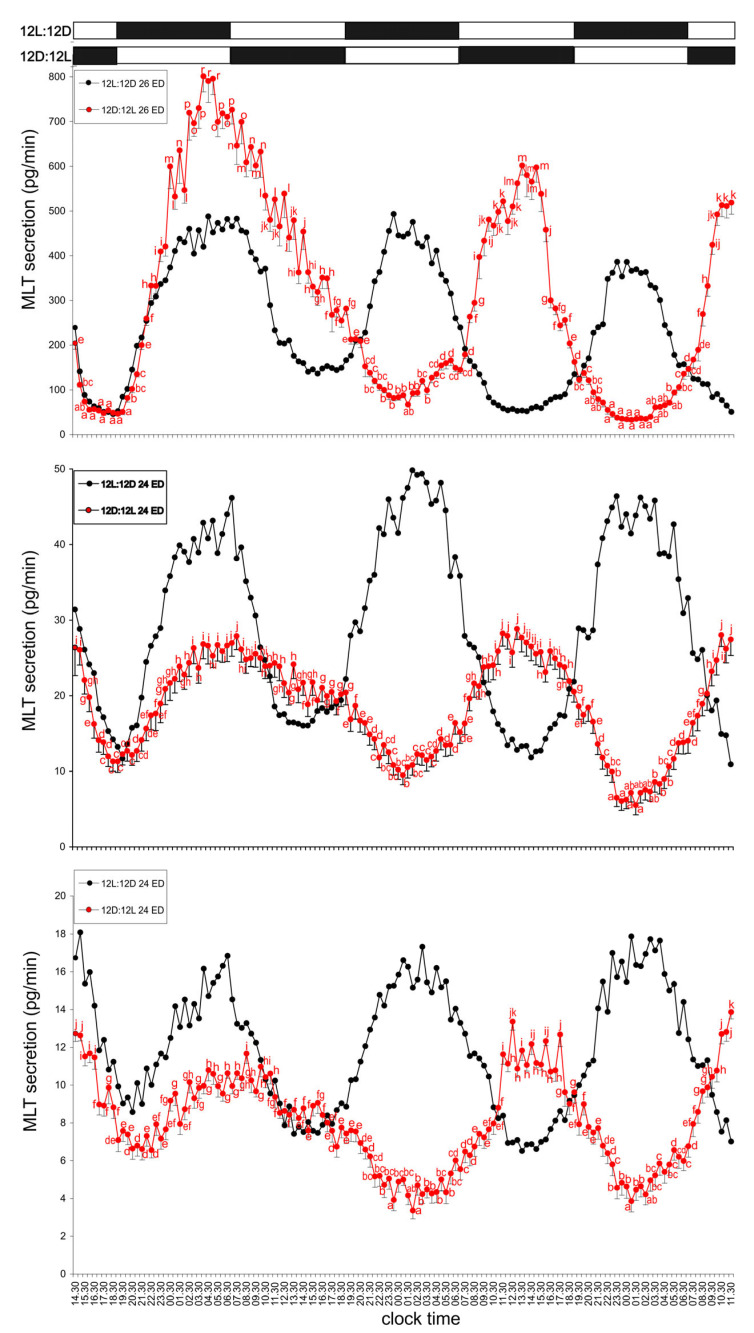
Comparison of melatonin (MLT) secretion (mean ± SEM) from the pineal organs of turkey embryos at ED 22–26, incubated for three days under either a 12L:12D light–dark cycle (photophase from 07:00 to 19:00) or a reversed 12D:12L cycle (photophase from 19:00 to 07:00). Different letters indicate significant differences between time points within each age group of explants incubated under the reversed 12D:12L cycle, while identical letters denote means that are not significantly different. MLT secretion in all age groups became entrained to the reversed light–dark cycle starting from the second day of incubation.

MLT secretion profiles in the pineal glands of embryos incubated under reversed light conditions (12D:12L; dark phase 07:00–19:00) were similar in pattern across all ages and differed only in hormone concentration (highest in ED 26, lowest in ED 22). MLT levels gradually increased from 20:00, reaching a broad peak between 01:00 and 11:00, and then declined to the lowest levels between 20:00 and 05:00. A second, shorter peak occurred between 10:00 and 16:00, followed by another low phase between 23:30 and 04:00. The amplitude of MLT secretion was approximately 10–12-fold in the oldest ED 26 embryos, 4.5-fold in the intermediate ED 24 group, and 3–4-fold in the youngest ED 22 (Figure 2).

#### 3.1.3. Group III—Three-Hour Light Exposure at Night

**Figure 3:**
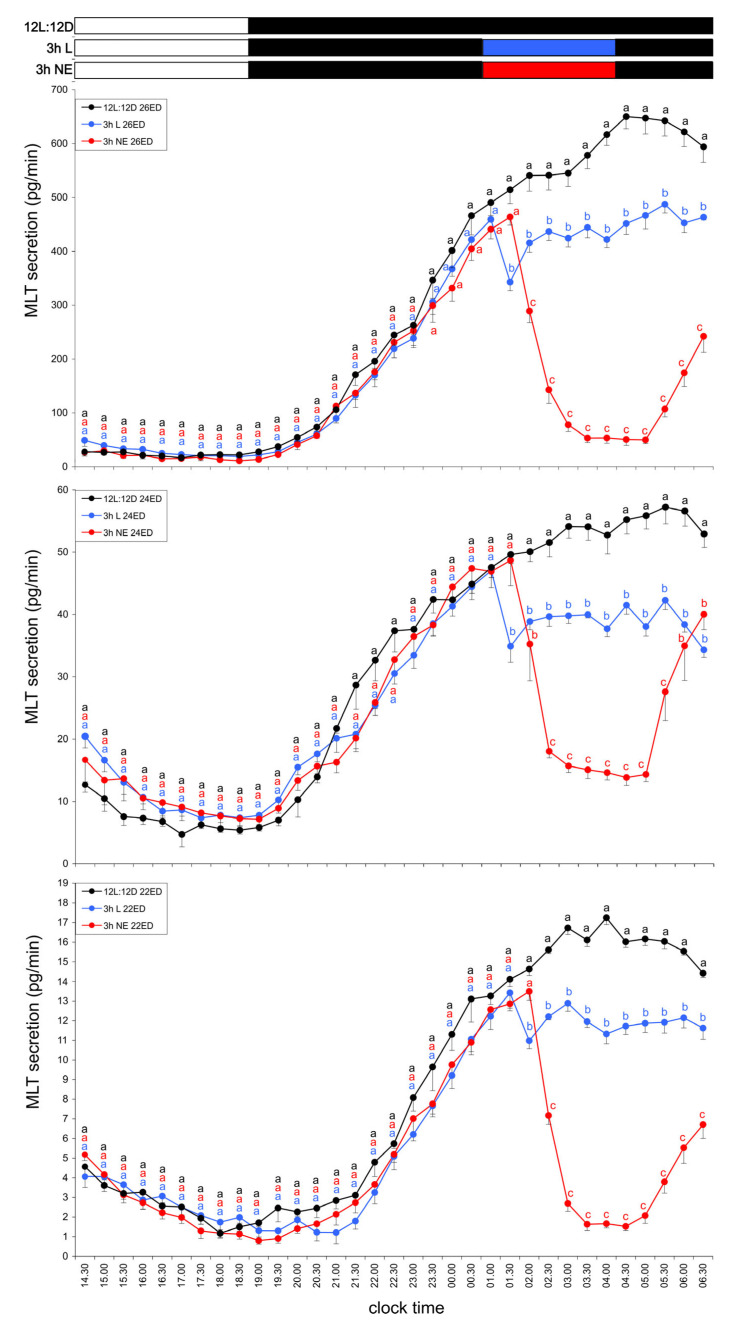
Secretion of melatonin (MLT; mean ± SEM) from pineal organs of turkey embryos at ED 22–26, incubated for one day under a 12L:12D cycle (photophase from 07:00 to 19:00). During the night (01:00–04:00), explants in the light (L) group were exposed to light at 100 lx, while explants in the NE group were incubated in medium containing norepinephrine (NE; 10 µM). Control (C) explants were maintained under standard dark conditions. Different letters indicate significant differences between treatment groups of the same age at the same time points; identical letters denote means that are not significantly different. Both light exposure and NE treatment significantly reduced MLT secretion, with the inhibitory effect of NE being markedly stronger than that of light.

A 3 h light pulse during the first night of incubation (01:00–04:00) caused a distinct transient decrease in MLT levels by approximately 30%. This suppression lasted 2–3 h, after which MLT levels rapidly returned to the elevated nocturnal values observed in Group I. The transient decline in MLT secretion was consistent across all embryonic stages (Figure 3).

### 3.2. Experiment II—Activity of the Endogenous Oscillator

#### 3.2.1. Group IV—24D Cycle (Continuous Darkness)

**Figure 4:**
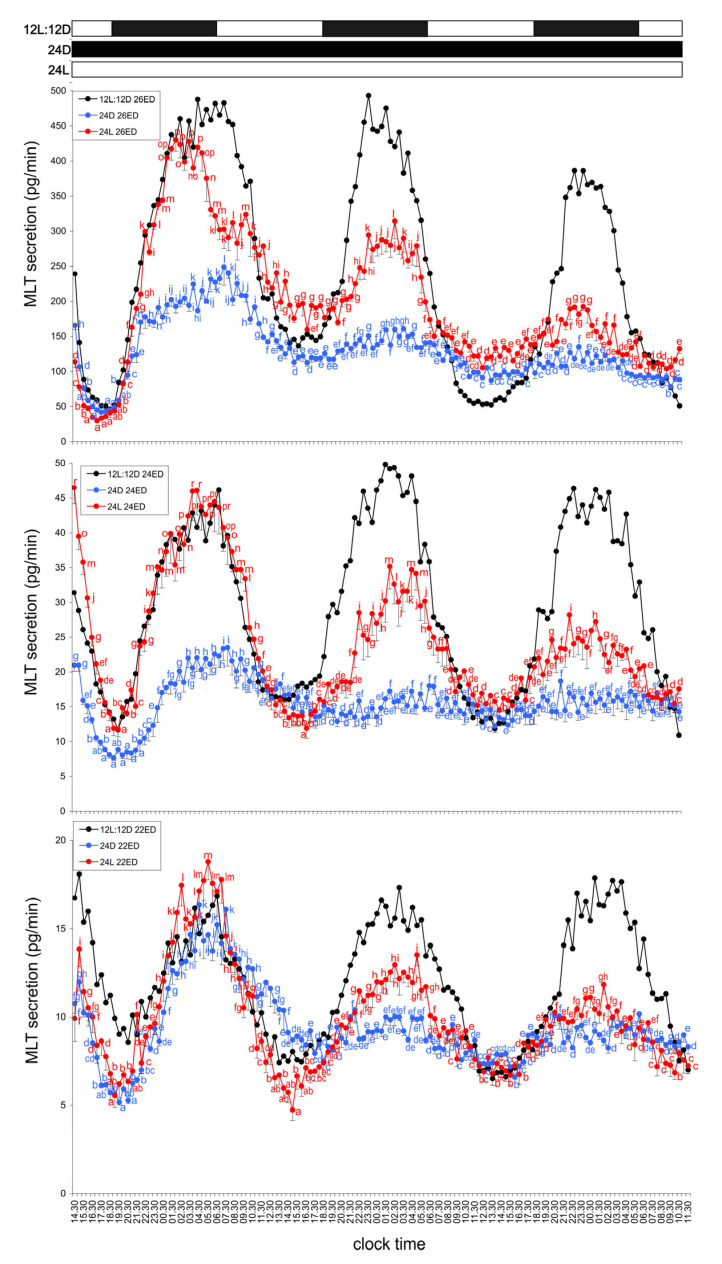
Comparison of melatonin (MLT) secretion (mean ± SEM) from the pineal organs of turkey embryos at ED 22–26, incubated for three days under a 12L:12D cycle (photophase from 07:00 to 19:00), continuous darkness, or continuous light. Different letters indicate significant differences between time points within each age group of explants incubated under continuous darkness or continuous light; identical letters denote means that are not significantly different. Note the rhythmic secretion of MLT from pineal organs maintained under both continuous darkness and continuous light.

Pineal glands from all turkey embryos incubated under continuous darkness secreted MLT rhythmically throughout the experiment. On the first day, MLT secretion increased after 19:00, reached a broad peak between 00:30 and 12:30, and then declined to its lowest level between 18:00 and 22:00. In ED 26 embryos, clear rhythmicity persisted during the second and third days, with MLT peaks between 23:30 and 05:00, approximately twofold lower than the first-day maximum. The amplitude of MLT secretion during the last two days was about twofold. In younger embryos (ED 24 and ED 22), only slight rhythmic fluctuations were detected during the last two days, with MLT levels 1.5–2 times lower than the first-day peak (Figure 4).

#### 3.2.2. Group V—24L Cycle (Continuous Light)

Pineal glands from all embryonic stages incubated under continuous illumination also secreted MLT rhythmically. Elevated MLT levels were observed each day between 01:00 and 10:00. The lowest levels on days 2 and 3 occurred between 01:00 and 08:00 but remained significantly higher than those recorded on day 1. The amplitude of MLT secretion on day 1 was approximately 3.5–4-fold and decreased to less than twofold during the following two days of incubation (Figure 4).

### 3.3. Experiment III—Effect of Norepinephrine

#### 3.3.1. Group VI—NE During the Second and Third Subjective Days

**Figure 5:**
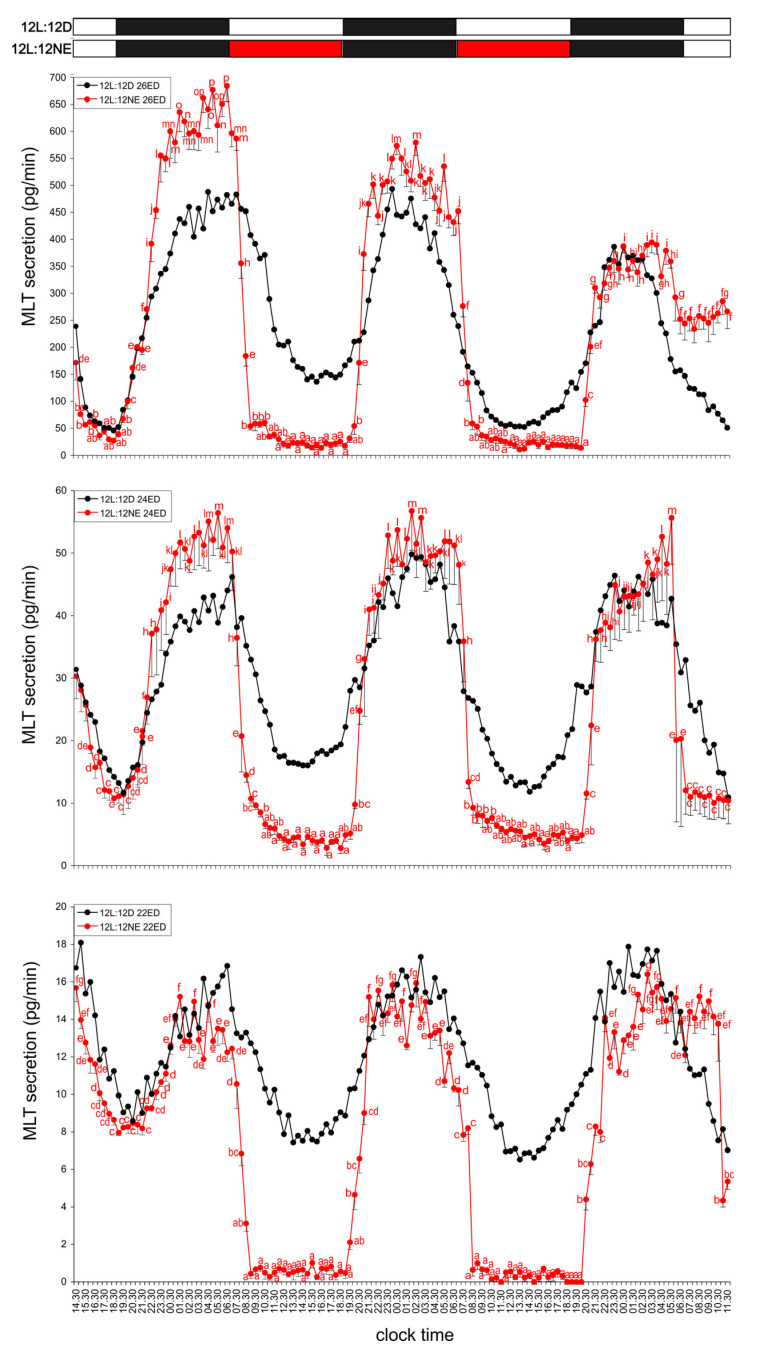
Comparison of melatonin (MLT) secretion (mean ± SEM) from the pineal organs of turkey embryos at ED 22–26 incubated for three days under a 12L:12D cycle (photophase from 07:00 to 19:00) or for one day under a 12L:12D cycle followed by two days in continuous darkness with norepinephrine (NE; 10 µM) added to the culture medium during the subjective day. Different letters indicate significant differences between time points within each age group of explants treated with NE; identical letters denote means that are not significantly different. MLT secretion in NE-treated explants exhibited a clear, high-amplitude daily rhythm under constant darkness.

Addition of NE to the culture medium during the second and third subjective days caused a rapid and pronounced decline in MLT levels between 07:00 and 19:00. In the youngest ED 22 embryos, NE reduced MLT secretion to values below the detection limit of the assay. Immediately after NE exposure, MLT levels in all embryos increased rapidly, returning to high nocturnal values similar within each age group across all nights of the experiment (Figure 5).

#### 3.3.2. Group VII—Three-Hour NE Exposure at Night

NE added to the culture medium from 01:00 to 04:00 during the first night of incubation significantly suppressed MLT secretion in all embryonic stages. Between 02:30 and 05:00, MLT levels decreased approximately sixfold in ED 26 embryos and fourfold in ED 24 and ED 22 embryos (Figure 3).

## 4. Discussion

**Figure 6:**
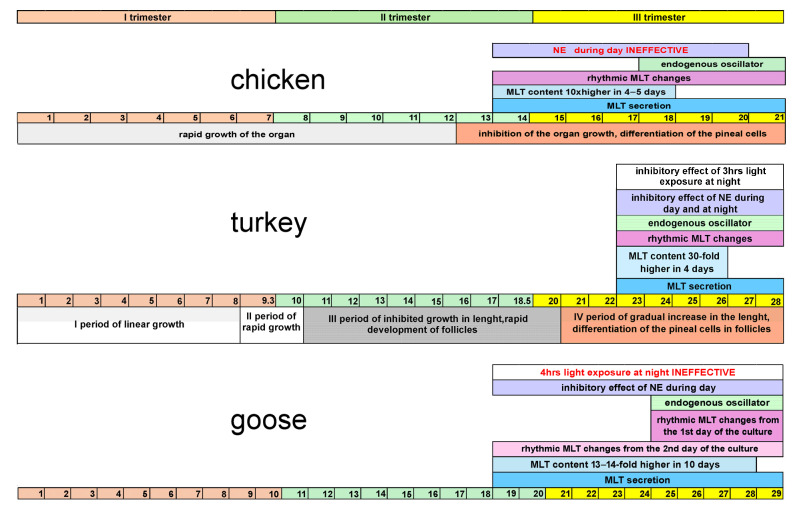
Comparative timing of measurable pineal melatonin (MLT) secretion in three avian embryo species. Turkey data: present study; chicken and goose: literature sources cited in the Discussion. The figure is provided for contextual comparison and was not part of the experimental design. Colored bars indicate the timing of key developmental milestones: onset of measurable MLT secretion, emergence of rhythmic MLT fluctuations, demonstration of an endogenous oscillator, and norepinephrine (NE) responsiveness. In turkey, all functional features—light sensitivity, circadian rhythmicity, and NE-mediated inhibition—coincide with the onset of measurable secretion at embryonic day 22 (ED 22) under the present experimental conditions.

This study showed that turkey pinealocytes secreted melatonin (MLT) in rapidly increasing amounts during late incubation. Under a 12L:12D cycle, the highest MLT concentrations from ED 26 explants were over 30-fold higher than those from ED 22, where levels were near the detection limit. In the superfusion culture and assay system, MLT release was first reliably measurable at ED 22; earlier stages were below detection, so ED 22 (six days before hatching) is treated as the onset of measurable secretion under these conditions (not necessarily the absolute biological onset). This timing accords with unpublished in vivo observations reporting detectable MLT and other methoxyindoles from ED 22. For comparison, measurable secretion in chicken is reported at ED 13–14—seven to eight days before hatching [31,32,36], although trace amounts have been noted as early as ED 10 under static 24 h incubation [26]. In goose, measurable secretion appears by ED 18 (11 days before hatching) both in vivo and in vitro [3,39]. Because incubation length differs among species (21 days in chicken, 28 in turkey, 29–30 in goose), staging by “trimesters” provides a clearer comparison: MLT secretion emerges near the end of the second trimester in goose and chicken [3,32], and slightly later—mid-final trimester—in turkey (Figure 6).

The present study revealed a strong photosensitivity of turkey embryonic pinealocytes, characterized by precise adjustment of MLT secretion profiles to different light conditions. Under 12L:12D, all examined stages displayed a clear day–night rhythm with ~4-fold nocturnal-to-daytime amplitude that remained stable across three days of culture. By comparison, goose embryos showed a gradual increase in amplitude from ~1.5-fold to ~2.5–3-fold over successive days, driven by a progressive rise in the nocturnal level [3]. After hatching, rhythm amplitude diverges further across species: ~40-fold in 12–14-week-old turkeys, whereas in geese it remains near the ~3-fold level, similar to late embryonic values [14,20].

Day–night differences in MLT release were evident from the first day of culture at all turkey stages maintained in 12L:12D. In chicken, day–night differences appear in cultures from ED 13–14, coincident with the onset of secretion [31]. In goose, rhythmicity arises later—about six days after secretion begins, from ED 24 onward [3]. The size of the nocturnal peak was also species-dependent: ED 26 turkeys exceeded ED 22 by ~30-fold; in chicken the ED 18 versus ED 14 difference was ~10-fold [32,36]; and in goose the peak rose ~13–14-fold across a 10-day interval [3]. Thus, turkey embryos showed the largest proportional increase over the shortest developmental window among the species examined.

All studied turkey embryos also adapted the MLT rhythm to a reversed dark–light cycle (12D:12L) beginning on the second day of culture. Nocturnal peaks aligned with the new dark phase, with amplitude slightly lower than under the natural light–dark cycle. Moreover, a 3 h nocturnal light pulse reduced MLT by ~30% with rapid recovery thereafter, indicating high acute photosensitivity. By contrast, a 4 h nocturnal light exposure did not suppress MLT in goose embryos [3] (Figure 6). These results are consistent with reports in 12-week-old turkeys, where light pulses suppress MLT regardless of timing within the night [14], and together suggest a robust photic control operating across a wider circadian window in turkey than in goose.

Incubation of pineal glands under continuous darkness or continuous light confirmed the presence of an active endogenous oscillator generating rhythmic MLT secretion in all turkey embryos. Rhythmic secretion persisted under both continuous darkness (DD) and continuous light (LL), with stronger and more regular oscillations in LL than in DD throughout the three-day experiment; the effect was most pronounced at ED 26 but remained discernible, though weaker, at ED 22. Similar LL > DD differences have been reported in 12-week-old turkeys [14]. The results indicate that the fundamental properties of the turkey pineal oscillator are already established at the onset of its activity in embryonic life and are maintained thereafter. In chicken and goose, endogenous rhythmicity emerges several days after MLT synthesis begins: ED 17–18 in chicken (≈4 days after onset) and ~6 days later in goose [31,32], where rhythms in embryos and even in 12-week-old birds attenuate after 1–2 days in vitro [3,20]. Taken together, across the three species the endogenous oscillator becomes clearly demonstrable during the latter part of incubation—the middle of the third trimester (Figure 6), with differences in timing and persistence that track each species’ developmental profile, coinciding with advanced pineal differentiation.

Adaptation of the pineal rhythm to the reversed dark–light cycle in turkeys began on the second day of incubation, similar to chickens and geese [3,31]. However, the first day differed markedly among species. In turkeys, the MLT profile during the first reversed cycle resembled that under the standard light–dark regime, with slightly lower and prolonged nocturnal levels extending into the following morning. This suggests that, in turkeys, the endogenous oscillator initially maintained the previously established rhythm pattern, temporarily overriding direct photic input. In contrast, in chickens and geese—where the oscillator develops later—the first day under reversed conditions produced either an immediate shift (chicken) or a transient abolition (goose) of rhythmic secretion [3,31].

Adrenergic control was already strong in turkey embryos. NE added during the subjective day (07:00–19:00 in DD) produced rapid and pronounced suppression of MLT at all ages, reducing secretion in the youngest group below the assay detection limit; levels recovered promptly to high nocturnal MLT values after NE removal. Overall, NE inhibition exceeded the effect of a light pulse, underscoring the potency of adrenergic input at this stage. Comparable daytime suppression has been documented in goose, duck, and older turkey preparations [3,13,20], whereas in ED 19 chicken embryos NE was ineffective during the day but reduced nocturnal MLT by ~50% [33]. The present data therefore indicate that functional adrenergic receptors—and possibly effective sympathetic influence—are in place in turkey by ED 22; this is further supported by the short 3 h nocturnal NE treatment, which evoked a rapid sixfold decline—far stronger than a light pulse of the same duration (Figure 3 and Figure 6).

In summary, all three functional characteristics of the turkey pineal gland—photosensitivity, endogenous oscillator activity, and NE responsiveness—were present from the first day of measurable secretion of MLT (ED 22) and became stronger with development. These features were more pronounced than in other avian species, where they appear at later, distinct stages. I report this earlier maturation in turkey as a descriptive finding; its cause cannot be determined from these data without parallel, standardized multi-species studies under identical conditions.

Although the molecular aspects of pineal development were not examined here, physiological maturity in turkey aligns with morphological maturity. ED 22 corresponds to the late histological stage of pineal growth (ED 20 to hatching), when follicles are fully developed and dominate the parenchyma, and follicular cells are morphologically differentiated [24]. In chicken, pinealocyte differentiation begins around ED 12 and coincides with the onset of melatonin secretion [22,25,26]. By ED 24 and ED 26 in turkey, the gland contains a greater number of larger—often branching—follicles with fully differentiated cells, consistent with the robust functional readouts.

Molecular markers were not quantified in this work. As context, increasing MLT output and consolidation of the rhythm are consistent with rising AANAT capacity and downstream ASMT/HIOMT activity [1,39,43,44,45]; growing NE sensitivity aligns with maturation of α_2_-adrenergic receptor expression and coupling; and the shift from damped to robust rhythms is compatible with the coordinated onset of core clock gene oscillations (*Per*, *Cry*, *Bmal1*, *Clock*) [29,30,40]. These links were not tested here; they are provided to guide focused future work that directly evaluates the proposed molecular timelines.

Superfusion culture method enables precise control of stimuli (e.g., light and NE) and dense sampling, making time-course effects clear. At the same time, it is a simplified model that lacks whole-body signals (such as circulating catecholamines and glucocorticoids) and may alter local paracrine interactions. I therefore treat estimates of light and NE responsiveness as conservative relative to in vivo conditions. It is reassuring that the core phenomena observed here—the emergence and light entrainment of the rhythm and NE-mediated inhibition—align with reports from intact preparations, which supports the interpretation while making the model’s boundaries explicit.

Overall, these findings fill a clear gap in understanding avian pineal development and its chronobiological regulation. They show that the embryonic turkey pineal reaches a functional state that permits light-dependent and neurochemical control of melatonin well before hatching. The study provides the first coherent characterization of secretion dynamics and regulatory features in turkey embryos, offering a practical framework for timing and assessing functional maturation in avian pineal tissue.

## 5. Conclusions

This study demonstrates that MLT secretion in the embryonic turkey pineal gland incubated in the superfusion culture and assay conditions employed could be reliably measured for the first time on embryonic day 22 (ED 22). From this stage onward, turkey pinealocytes exhibited all key features of post-hatching pineal function—clear photosensitivity, an active endogenous oscillator, and strong responsiveness to NE-mediated inhibition. The presence of these mechanisms from the onset of MLT secretion indicates that the cellular and molecular systems underlying circadian control are established before hatching. These results suggest that the essential functional characteristics of pineal regulation are evident by ED 22, i.e., well before hatching. Secretion at earlier stages cannot be excluded. To pinpoint the onset of measurable secretion and clarify its mechanistic basis, finer-grained staging under identical conditions will be needed.

## Data Availability

The data supporting the findings of this study are contained within the article (figures). Raw time-series data and radioimmunoassay output files are available from the corresponding author upon reasonable request.
